# Anomalous Dispersion in Reflection and Emission of Dye Molecules Strongly Coupled to Surface Plasmon Polaritons

**DOI:** 10.3390/nano14020148

**Published:** 2024-01-09

**Authors:** Md Golam Rabbani Chowdhury, Leila Hesami, Kanij Mehtanin Khabir, Shamaar R. Howard, Md Afzalur Rab, Natalia Noginova, Mikhail A. Noginov

**Affiliations:** Center for Materials Research, Norfolk State University, Norfolk, VA 23504, USA; l.hesami@spartans.nsu.edu (L.H.); k.khabir@spartans.nsu.edu (K.M.K.); s.r.howard103100@spartans.nsu.edu (S.R.H.); m.a.rab@spartans.nsu.edu (M.A.R.); nnoginova@nsu.edu (N.N.); mnoginov@nsu.edu (M.A.N.)

**Keywords:** surface plasmon polariton, dispersion, strong coupling, dye molecules

## Abstract

We have studied dispersion of surface plasmon polaritons (SPPs) in the Kretschmann geometry (prism/Ag/dye-doped polymer) in weak, intermediate, and ultra-strong exciton–plasmon coupling regimes. The dispersion curves obtained in the reflection experiment were in good agreement with the simple model predictions at small concentrations of dye (Rhodamine 590, Rh590) in the polymer (Poly(methyl methacrylate), PMMA). At the same time, highly unusual multi-segment “staircase-like” dispersion curves were observed at extra-large dye concentrations, also in agreement with the simple theoretical model predicting large, small, and negative group velocities featured by different polariton branches. In a separate experiment, we measured angular dependent emission of Rh590 dye and obtained the dispersion curves consisting of two branches, one nearly resembling the SPP dispersion found in reflection and the second one almost horizontal. The results of our study pave the road to unparalleled fundamental science and future applications of weak and strong light—matter interactions.

## 1. Introduction

Nanophotonics. Nanophotonics offers scientists and engineers limitless opportunities that have changed the frontier of optics forever. It enabled squeezing of light into a nanoscale dimension, leading to numerous applications including photodetectors [1], nanoantennas [2], nanolasers [3], subdiffraction microscopy [4], and biomedical engineering [5].

Plasmonics. A subfield of nanophotonics is plasmonics [6], which is the study of generation, manipulation and detection of localized surface plasmons (LSPs) and propagating surface plasmons (SPs) (or surface plasmon polaritons (SPPs)). When light shines onto a metal–dielectric interface, it interacts with loosely bound free electrons and, under the right circumstances, the electromagnetic surface wave can couple with collective oscillation of electron plasma, giving rise to quasi-particles known as propagating SPs or SPPs. They can be viewed as electromagnetic excitations propagating at the interface between a dielectric and a conductor, evanescently confined in the perpendicular direction [7], and (often) having high local fields. Applications of SPs include subwavelength microscopy [8] and lithography beyond the diffraction limit [9]. Other applications are photonic data storage [10], light generation [11,12], bio-photonics [13], solar cells [14], routers [15], and optical tweezing [16].

Metamaterials. Metamaterials are artificially fabricated materials with properties which are not achievable in naturally occurring materials [17]. Often these tailored materials are made of arrays of basic building blocks, such as split ring resonators [18], to achieve immense control over electromagnetic waves, which enables, e.g., negative index of refraction [19], hyperbolic dispersion [20], hyperlens [21], superlens [22], and invisibility cloaking [23]. It is not always the inherent materials’ properties, but rather precise shapes, geometry, size, orientation, and arrangement of metamaterials’ building blocks that give metamaterials their unique properties. Control of light–matter interaction using metamaterials and nanostructures is a hot topic and a large platform for contemporary fundamental and engineering research.

**Figure 1:**
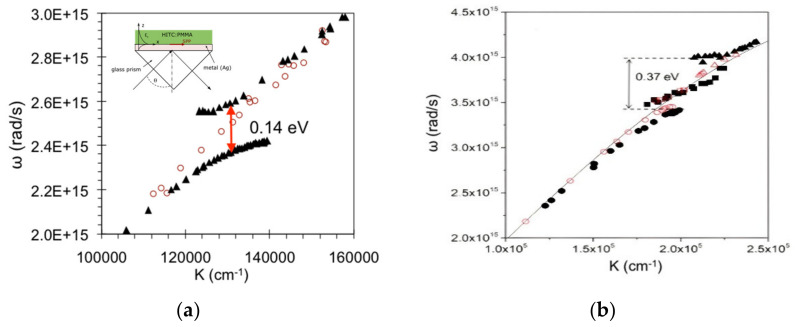
(**a**) Surface plasmon polariton (SPP) dispersion curve measured when the PMMA polymeric film is undoped (empty circles) and doped with HITC dye (solid black triangles), resulting in one avoided crossing or Rabi splitting. (**b**) Dispersion in PMMA doped with R6G dye. Two open gaps resulted from one absorption peak and a shoulder. Adopted from Refs. [37,38], respectively.

Strong Coupling. Let us consider two interacting subsystems consisting of, e.g., exciton and plasmon with identical or close energies. If the rate of the energy relaxation or dephasing is larger than the rate of the energy transfer between two constituents, the system is said to be in the weak coupling regime. This affects the relaxation rates in the system but not the eigenvalues of the energy states. On the other hand, if the interaction between two constituents is strong (energy transfer is faster than the relaxation rates), the excited energy states shared by two subsystems are becoming hybridized, and their energy eigenvalues and spectral positions change, as well as the relaxation lifetimes. This regime is known as the strong coupling regime. It features splitting and avoided crossing of the dispersion curves [24,25], whose magnitude (Rabi splitting) is proportional to the square root of the exciton’s concentration (Figure 1a). If the energy shifts of hybridized states become comparable to the energy values of individual unperturbed constituents, the system is said to be in the ultra-strong coupling regime [26,27].

The hybrid energy states change the electronic structure of the molecule, achieving a remarkable degree of control over material properties, such as the modification of chemical reaction rates [28], electrical conductivity [29], surface potentials [30], and control over phase transitions [31]. Intriguing fundamental science and applications of strong coupling inspired the present study.

The dispersion curves of surface plasmon polaritons (SPPs) excited in the Kretschmann geometry (high-index prism/plasmonic metal/low-index dielectric) are very well known in the literature [32,33,34,35] (Figure 1a,b).

As discussed above, if the dielectric has an absorption band of (quantum) emitters, which are strongly coupled with SPPs, this results in the avoided crossing and the Rabi splitting of the dispersion curve [36,37] (Figure 1a). Two absorption bands (or a band and a shoulder) reportedly result in three dispersion branches and two open gaps [38] (Figure 1b). The coupling strength, proportional to the square root of the concentration of excitons or other emitters, is expected to affect the magnitude of the (single) Rabi splitting, but not the spectral positions or the very existence of multiple dispersion branches and Rabi energy gaps [25], as experimentally shown below.

The uniqueness of strong coupling phenomena, leading to fundamental science and matchless applications, motivated the study reported below. Although reflection and emission are among the most studied strong coupling phenomena, we have observed highly unusual dispersion behavior for reflection, which was strongly different from emission and, according to our knowledge, never reported in the literature.

## 2. Experimental Methods and Results

### 2.1. Sample Fabrication

**Figure 2:**
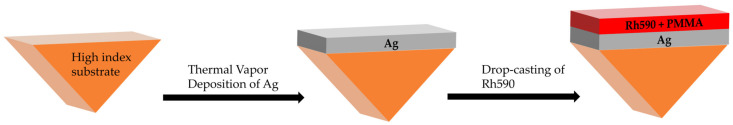
Chart Flow: Preparation of the Kretschmann geometry samples (high index prism/plasmonic metal/low index dielectric).

Experimentally, silver films (~40 nm) were deposited onto the hypotenuse surfaces of high index right angle prisms (15 mm × 15 mm), and dye-doped polymer (Rh590:PMMA) of various dye concentrations was coated on silver (Figure 2). The concentrations of Rhodamine dye ranged between 0 g/L (pure PMMA) and 1260 g/L (pure Rh590). The dye-doped polymeric solution was dissolved in dichloromethane (DCM) and drop-casted onto the silver film. The thicknesses of the dye-doped polymeric films were in the range of d = 2~3 μm. The Nano 36 Thermal Evaporation Deposition System (from Kurt J Lesker, Jefferson Hills, PA, USA) was used for deposition of Ag films. The thickness of the films was measured using the stylus DekTak XT profilometer from Bruker (Billerica, MA, USA). See Methods for details.

### 2.2. Dispersion Measurement in the Reflection Experiment

**Figure 3:**
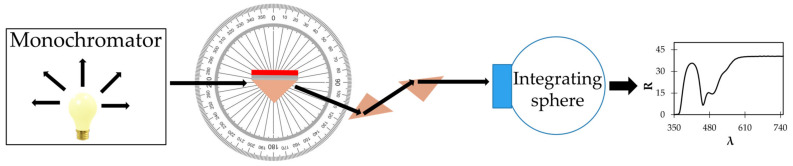
(**Left**): Schematic of the spectrophotometer set-up used to record reflectance spectra in the Kretschmann geometry. (**Right**): Typical reflection spectrum of the Kretschmann geometry sample.

For the reflection experiment, we used the spectrophotometer, and the samples were illuminated using p-polarized light. The setup had a rotating stage that allowed us to rotate the prism and take spectral tens of measurements at different angles. The reflected light was guided, using several other prisms, to the integrating sphere, yielding reflection spectra with one or multiple dips (Figure 3). The wavelength positions of these dips were then measured and converted into dispersion curves *k* (ω).

**Figure 4:**
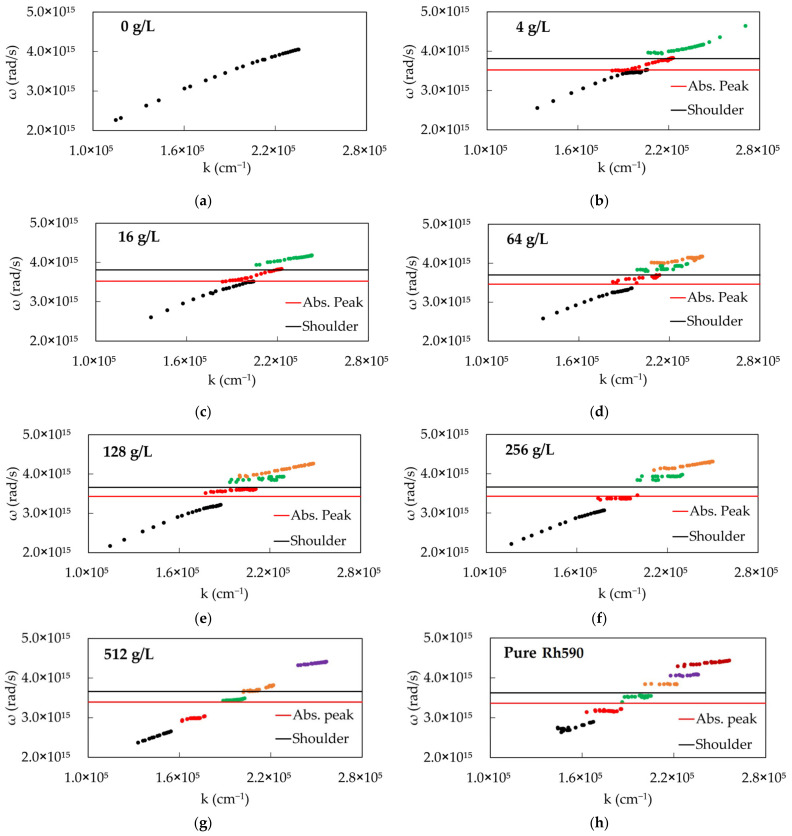
Dispersion curves for SPPs coupled with dye-doped polymer (Rh590:PMMA) in the Kretschmann geometry, at dye concentrations equal to (**a**) 0 g/L (pure PMMA), (**b**) 4 g/L, (**c**) 16 g/L, (**d**) 64 g/L, (**e**) 128 g/L, (**f**) 256 g/L, (**g**) 512 g/L and (**h**) 1260 g/L (pure Rh590). Red horizontal lines: spectral maxima of Rh590 absorption; Black horizontal lines: Rh590 absorption shoulder.

The dispersion curves for several dye concentrations exhibited several different types of behavior, as can be seen in Figure 4. With no dye, there is only one dispersion curve without splitting (Figure 4a). At relatively low concentrations, the dispersion curves had three branches with two Rabi splittings (Figure 4b,c). According to Ref. [38] and the theoretical model below, this type of behavior is characteristic of samples having two absorption bands or an absorption band and a shoulder, which are denoted by black and red horizontal lines in Figure 4b–h. At the same time, the dye having only one absorption band is characterized by two branches of the dispersion curves separated by the Rabi energy gap [37], Figure 1a. At high dye concentrations, ≥128 g/L, the dispersion curves (Figure 4e–h) consisted of multiple (up to six) branches with five Rabi splittings. This highly unusual “staircase-like” behavior will be discussed below. At intermediate dye concentrations, such as 64 g/L, the dispersion curves consisting of several branches demonstrated a transitional behavior from low to high concentrations (Figure 4d).

Overall, the number of dispersion branches grows with increasing dye concentration (Figure 4). However, no correlation between the thickness of the Rh590:PMMA film *d* and the SPP dispersion curves has been observed. Furthermore, the reflection spectra calculated using (i) the well-known analytical formula [35] (assuming the dielectric medium on top to be infinitely thick, *d* = ∞) and (ii) using the finite-element method (FEM) solver, COMSOL Multiphysics, (assuming the dielectric medium to be *d* = 2 μm thick), are in very good agreement with each other. Therefore, we conclude that in this particular study, the effect of the *d* = 2 μm thick Rh590:PMMA film is the same as the effect of the infinitely thick film, *d* = ∞.

### 2.3. Dispersion Curves Obtained from the Emission Experiment

**Figure 5:**
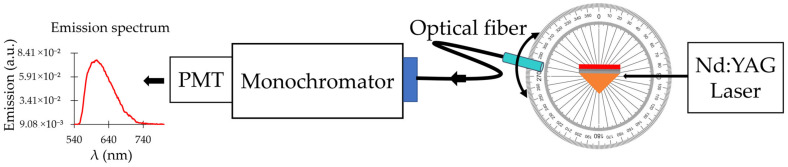
(**Left**): Typical emission spectrum of Rh590. (**Right**): Schematic of the experimental setup for spontaneous emission study.

**Figure 6:**
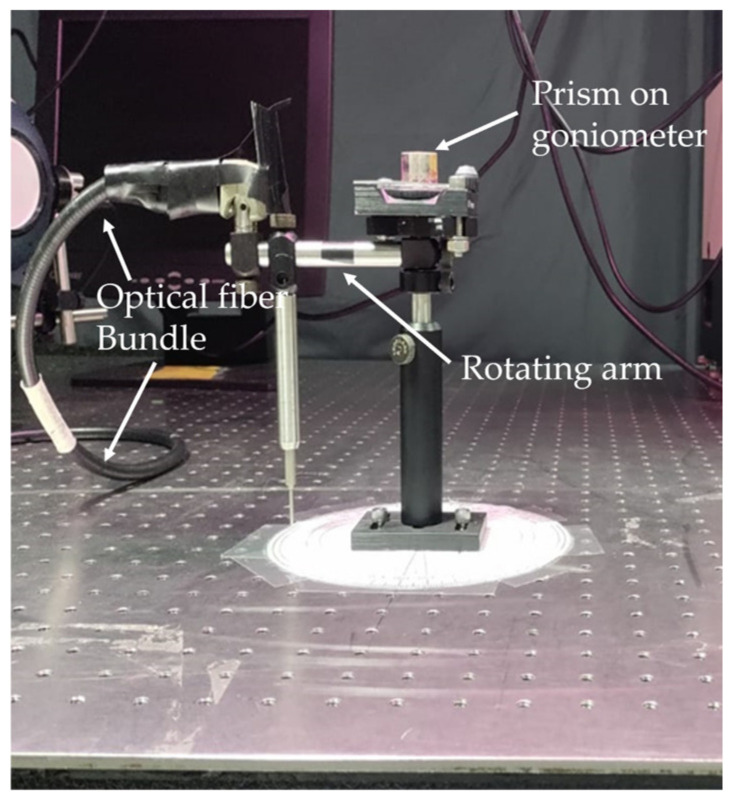
Photograph of the experimental setup showing the prism, the prism holder and the shaft holding and rotating the optical bundle.

In the next experiment, we measured the dispersion curve in the emission experiment and compared it with the result obtained in the reflection experiment. In the emission setup (Figure 5 and Figure 6), we illuminated the prism samples with Q-switched frequency doubled Nd:YAG laser (t_pulse_ = 12 ns and λ = 532 nm). The prism placed on the rotation stage was rotated to the angle at which the SPP at 532 nm was excited. Once the prism was fixed at that critical angle, a shaft holding an optical fiber bundle was placed at different angular positions around the prism to collect spontaneous emission of the dye molecules and deliver it to the entrance slit of the monochromator (MS 257 from Oriel/Newport) (Figure 5). The collection of spontaneous emission spectra measured at different angular positions of the optical bundle was analyzed to obtain the emission dispersion curves at different dye concentrations.

**Figure 7:**
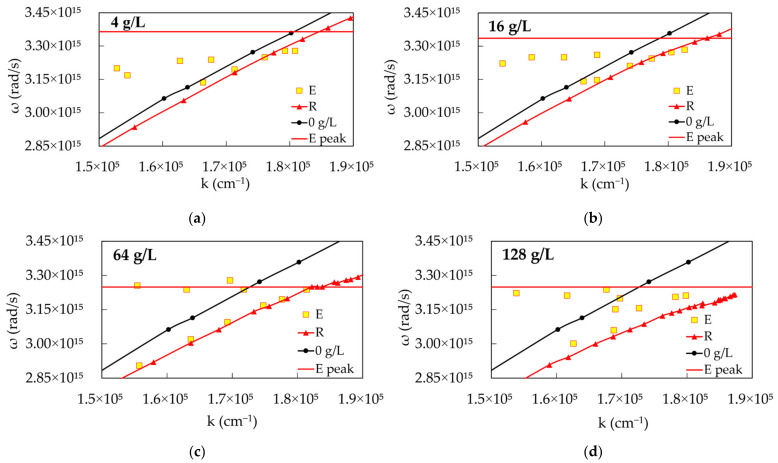
The dispersion curves obtained from the emission study for the dye concentrations (**a**) 4 g/L, (**b**) 16 g/L, (**c**) 64 g/L and (**d**) 128 g/L, compared to those obtained in the reflection experiments. Yellow squares with red borders: dispersion curves obtained from emission; Red line with triangles: lower polariton branch obtained in the reflection experiments; Black line with circles: dispersion curves obtained from reflection of PMMA with no dye; Red horizontal line: Spectral position of the maximum emission intensity measured with spectrofluorometer.

The emission study resulted in dispersion curves which strongly deviated from those obtained in the reflection experiments, compared in Figure 4b–e and Figure 7a–d. The dispersion curves obtained at dye concentrations 4 g/L, 16 g/L, 64 g/L and 128 g/L are similar to each other (Figure 7a–d). Therefore, we describe in detail the dispersion curves obtained at 64 g/L (Figure 7c), having in mind that the other dispersion curves are qualitatively similar.

In Figure 7a–d, yellow squares with red borders denote dispersion curves obtained from emission experiments, red lines with triangles denote lower polariton branches obtained from the reflection experiments, black lines with circles denote dispersion curves obtained from reflection spectra of PMMA with no dye, and the red horizontal line is the maximum emission intensity measured with the spectrofluorometer (Fluorolog 3 from Jobin Yvon Horiba).

A close inspection reveals that the emission dispersion has two branches: one of them follows the lower polariton branch obtained from the reflection experiment, while another branch (likely due to the molecules poorly coupled to SPPs) follows the red horizontal line. Another observation is that, with increasing dye concentration, both reflection and emission dispersion curves deviate more and more from the dispersion curves measured with PMMA without dye (black traces with circular characters).

## 3. Theoretical Modeling

**Figure 8:**
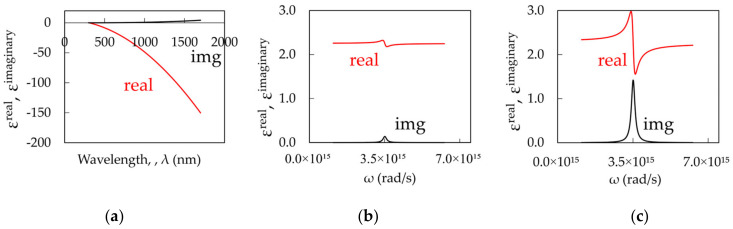
(**a**) Real and Imaginary parts of the dielectric permittivity of Ag calculated using the Drude model (Equation (1), [39]). The dielectric permittivity of dye-doped polymer calculated using a single Lorentzian model for (**b**) low (k_abs_^max^ = 10^4^ cm^−1^) and (**c**) high (k_abs_^max^ = 10^5^ cm^−1^) dye concentrations (Equation (2), [40]).

The anomalous behavior of experimental dispersion curves can be modeled using the concept of strong coupling between surface plasmons and excitons. The dielectric permittivity of Ag was calculated using the Drude model (Equation (1), Figure 8a, [39]), and the dielectric permittivity of dye-doped PMMA was modeled using one (Equation (2), Figure 8b, [40]) or two Lorentzian functions (Equation (3), Figure 8c, [40]);

(1) $$\hat{\varepsilon }\left(\omega \right)=\varepsilon_{\infty }-\frac{\omega_{p}^{2}}{\omega^{2}+i\Gamma_{D}\omega },$$

(2) $$\hat{\varepsilon }\left(\omega \right)=\varepsilon_{\infty }+\frac{A_{1}}{E_{1}^{2}-{\left(\hbar \omega \right)}^{2}-i\gamma_{1}\left(\hbar \omega \right)},$$

(3) $$\hat{\varepsilon }\left(\omega \right)=\varepsilon_{\infty }+\frac{A_{1}}{E_{1}^{2}-{\left(\hbar \omega \right)}^{2}-i\gamma_{1}\left(\hbar \omega \right)}+\frac{A_{2}}{E_{2}^{2}-{\left(\hbar \omega \right)}^{2}-i\gamma_{2}\left(\hbar \omega \right)},$$

where, $\varepsilon_{\infty }$ is the offset of the real part of the dielectric constant, $\omega_{p}$ is the plasma frequency of the metal, $\Gamma_{D}$ is the effective damping constant for metal, ω is the optical frequency, $A_{1},A_{2}$ are the oscillator strengths for the Lorentzian absorbers, $E_{1},E_{2}$ are the photon resonant energies, $\gamma_{1},\gamma_{2}$ are the decay rates in a dielectric and ℏ is the reduced Planck constant.

**Figure 9:**
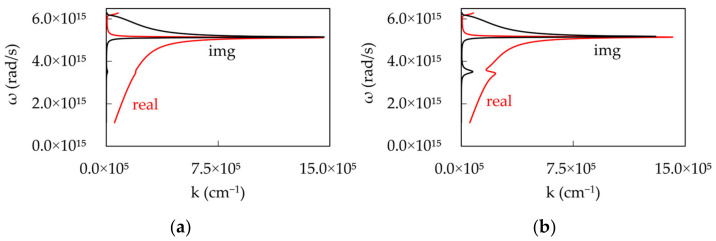
Real and Imaginary parts of the SPP dispersion calculated for single Lorentzian absorptions at (**a**) low (k_abs_^max^ = 10^4^ cm^−1^) and (**b**) high (k_abs_^max^ = 10^5^ cm^−1^) dye concentrations.

Figure 8b depicts dielectric permittivity of dye-doped polymer at a relatively small Lorentzian absorption, k_abs_^max^ = 10^4^ cm^−1^, while Figure 8c shows dielectric permittivity at a large Lorentzian absorption, k_abs_^max^ = 10^5^ cm^−1^. The dispersion of the surface plasmon polaritons (SPPs) propagating at the metal–dielectric interface can be found using Equation (4) [35].

(4) $$k_{SPP}=\frac{\omega }{c}\sqrt{\frac{\varepsilon_{m}.\varepsilon_{d}}{\varepsilon_{m}+\varepsilon_{d}}},$$

where, $\varepsilon_{m}$ and $\varepsilon_{d}$ are the relative dielectric permittivities for the metal and the dielectric, respectively. The dispersion curves calculated for single Lorentzian absorptions at low and high dye concentrations are depicted in Figure 9a,b.

It should be noted that, at low dye concentration, the real and imaginary parts of the dispersion plots show characteristics minute “humps”, which become significantly larger at high dye concentrations.

### Strong Coupling between an Exciton and a Plasmon

Strong coupling between a single exciton and a surface plasmon polariton can be described by a 2 × 2 “Hamiltonian-like” equation [41], Equation (5).

(5) $$\left(\begin{array}{cc} \omega_{SPP} & ∆\\ ∆ & \omega_{Ex} \end{array}\right)\left(\begin{array}{c} \alpha_{SPP}\\ \alpha_{Ex} \end{array}\right)=\omega \left(\begin{array}{c} \alpha_{SPP}\\ \alpha_{Ex} \end{array}\right),$$

where, $\omega_{SPP}$ is the surface plasmon polariton frequency, $\omega_{Ex}$ is the frequency of not interacting excitons and plasmons, ∆ is the coupling strength factor, $\alpha_{SPP}$ and $\alpha_{Ex}$ are the polariton mixing coefficients and ω is the surface plasmon (light) frequency.

The eigenfrequencies $\omega^{+}$ and $\omega^{-}$ of the hybridized upper and lower polariton branches can be found using Equations (6) and (7).

(6) $$\left|\begin{array}{cc} \omega_{SPP}-\omega & ∆\\ ∆ & \omega_{Ex}-\omega \end{array}\right|=0$$

and

(7) $$\omega^{\pm }=\frac{\omega_{SPP}+\omega_{Ex}}{2}\pm \frac{\sqrt{{\left(\omega_{SPP}-\omega_{Ex}\right)}^{2}+4{∆}^{2}}}{2},$$

**Figure 10:**
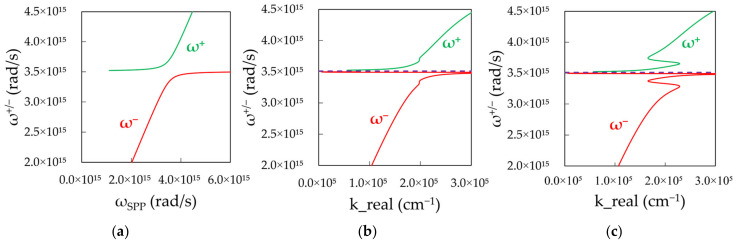
(**a**) Two polaritonic branches (upper and lower) plotted in terms of frequency versus frequency. Calculated dispersion in terms of ω versus *k* for (**b**) low, k_abs_^max^ = 10^4^ cm^−1^, and (**c**) high, k_abs_^max^ = 10^5^ cm^−1^, dye concentrations.

Figure 10a shows these frequencies plotted as the function of SPP frequency. The calculated curves have two expected branches and the Rabi frequency splitting. To plot these dispersion curves in terms of ω versus *k*, we combine Figure 10a with Figure 9a,b resulting in Figure 10b,c calculated for low and high dye concentrations. One can see that, at the low dye concentration, the calculated dispersion curve (Figure 10b) is in good qualitative agreement with the experimental one (Figure 1a). At the same time, the high concentration result shows highly unusual dispersion curves (Figure 10c), never reported in the literature, consisting of multiple branches with high, low, and negative group velocities.

**Figure 11:**
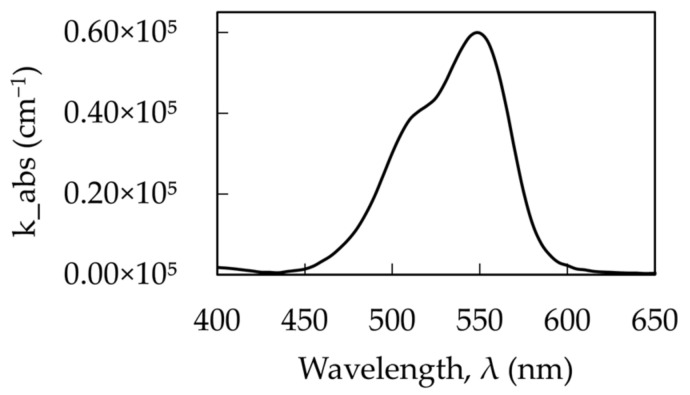
Typical absorbance spectrum of Rh590 with the absorption peak and the shoulder.

**Figure 12:**
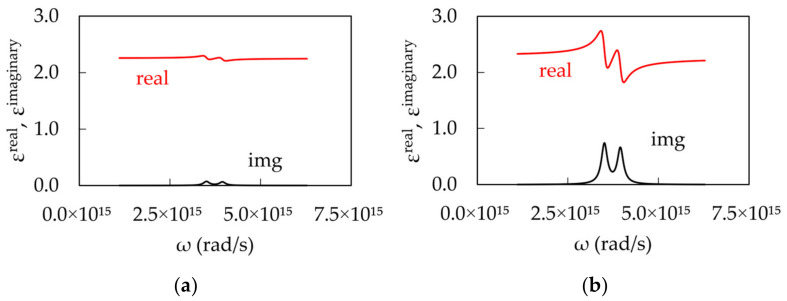
Real and Imaginary parts of the dielectric permittivity of dye doped polymer calculated using double-Lorentzian model for (**a**) low and (**b**) high dye concentrations, Equation (3) [40].

The dispersion curves above were calculated for a single Lorentzian oscillator. However, the absorption band of Rh590 consists of an absorption peak and a shoulder (Figure 11). Consequently, this absorption can be treated as a superposition of partly overlapping Lorentzian bands. The corresponding dielectric permittivities of dye-doped polymer, calculated using Equation (3) for small and large Rh590 dye concentrations, are depicted in Figure 12a,b.

**Figure 13:**
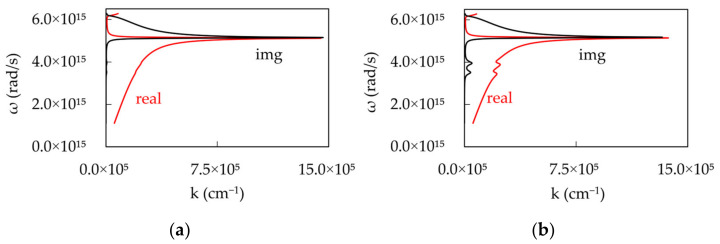
Real and Imaginary parts of the SPP dispersion for (**a**) low and (**b**) high dye concentrations resulting from the coupling of a surface plasmon with two excitons (two Lorentzians in the absorption spectra).

The dispersion curves of SPPs calculated (using Equations (1), (3) and (4)) for small and large double-Lorentzian absorption are depicted in the Figure 13a,b. It should be noted that, at low dye concentrations, the dispersion curves show minute “double hump” features, which become more visible at high dye concentrations.

The strong coupling between two excitons and one plasmon can be modeled by a 3 × 3 “Hamiltonian-like” equation [41,42] as shown below, (Equation (8)).

(8) $$\left(\begin{array}{ccc} \omega_{SPP} & ∆ & ∆\\ ∆ & \omega_{Ex1} & 0\\ ∆ & 0 & \omega_{Ex2} \end{array}\right)\left(\begin{array}{c} \alpha_{SPP}\\ \alpha_{Ex1}\\ \alpha_{Ex2} \end{array}\right)=\omega \left(\begin{array}{c} \alpha_{SPP}\\ \alpha_{Ex1}\\ \alpha_{Ex2} \end{array}\right),$$

where, ∆ is the strength of coupling between surface plasmon and two excitons (assumed to be the same for both excitons); $\alpha_{SPP}$, $\alpha_{Ex1}$ and $\alpha_{Ex2}$ are the polariton mixing coefficients; $\omega_{SPP}$, $\omega_{Ex1}$, $\omega_{Ex2}$ and ω represent the surface plasmon energy, the exciton energies and the polariton energy, respectively.

The eigenvalues for the polariton angular frequencies can be deducted by solving the following determinant equation.

(9) $$\left|\begin{array}{ccc} \omega_{SPP}-\omega & ∆ & ∆\\ ∆ & \omega_{Ex1}-\omega & 0\\ ∆ & 0 & \omega_{Ex2}-\omega \end{array}\right|=0$$

**Figure 14:**
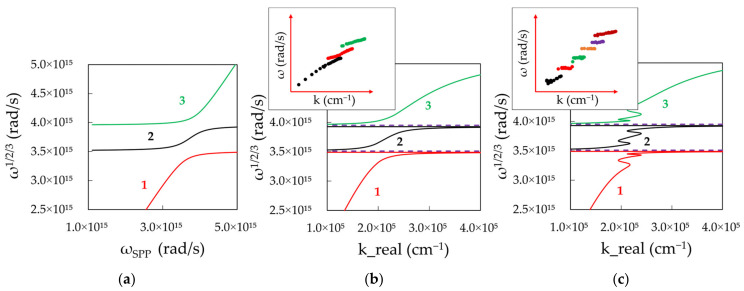
(**a**) Three polaritonic branches (upper, lower and middle) plotted in terms of frequency versus frequency. Calculated dispersion in terms of ω versus *k* for (**b**) low, k_abs_^max^ = 5.84 × 10^3^cm^−1^, and (**c**) high, k_abs_^max^ = 5.89 × 10^4^ cm^−1^, dye concentrations. Inset of (**b**): experimental dispersion curves at small concentration of Rh590, 16 g/L (Figure 4c). Inset of (**c**): experimental dispersion curves at large concentration of Rh590, 1260 g/L (Figure 4h).

The solution of this equation has three polaritonic branches (upper, lower and middle) plotted in Figure 14a in terms of frequency versus frequency. To calculate the same dispersion curve in terms of ω versus *k*, we combine Figure 14a with Figure 13a,b resulting in the double-Lorentzian dispersion for low (k_abs_^max^=5.84 × 10^3^cm^−1^, Figure 14b) and high (k_abs_^max^ = 5.89 × 10^4^ cm^−1^, Figure 14c) dye concentrations. The low concentration result (Figure 14b) demonstrated good qualitative agreement with the expected experimental behavior, which was observed above and in Ref. [38] (Figure 1b), whereas the high concentration result manifested anomalous and unprecedented dispersion that qualitatively resembles our experimental observations (Figure 4g,h).

Note that the dispersion curves depicted in Figure 14c are highly sensitive to absorption, coupling and other parameters. The purpose of this study was to show that a reasonable qualitative agreement of the experimental and theoretical results is possible. The detailed study of the anomalous dispersion curves is the subject of the future work.

## 4. Discussion

We studied dispersion at weak and strong coupling of SPPs in a prism geometry and Rh590 dye, featuring two absorption bands (main peak and a shoulder), and found two-to-five Rabi-like energy splits and three-to-six dispersion branches. Future studies will call for easier schemes involving excitons (dyes) with only one absorption band. However, finding such dyes may be a challenge. On the opposite, high complexity side, one can mix two dyes, with one or two absorption bands each, resulting in systems with two, three or four excitons. The challenge of availability of such dyes can be mitigated by varying concentration of both donors (high energy molecules) and acceptors (low energy molecules).

The experimental dispersion curves can be fitted using (i) the Hamiltonian-like model developed in this study, (ii) the analytical model for reflection in the Kretschmann geometry [35] as well as (iii) numerical COMSOL Multiphysics simulations. The latter has an advantage of calculating electric and magnetic field distributions in both metal and dye-doped dielectric layers at both low and high dye concentrations.

The experimental and theoretical studies above are the subject of separate research to be published elsewhere.

## 5. Summary

To summarize, we have studied dispersion of surface plasmon polaritons (SPPs) in the Kretschmann geometry (prism/Ag/dye-doped polymer) in weak, intermediate and ultra-strong exciton-plasmon coupling regimes. The dispersion curves obtained in the reflection experiment were in good agreement with the simple model predictions at small concentrations of dye (Rh590) in the polymer (PMMA). At the same time, highly unusual multi-segment “staircase-like” dispersion curves were observed at extra-large dye concentrations, also in agreement with the simple theoretical model predicting large, small and negative group velocities featured by different polariton branches. To the best of our knowledge, this result has never been reported in the literature.

In a separate experiment, we measured angular-dependent emission of Rh590 dye and obtained the corresponding dispersion curves (all consisting of two branches), one nearly resembling the SPP dispersion found in reflection and the second one almost horizontal. The behavior of these relatively simple dispersion curves is a subject of a future study to be published elsewhere.

The results of our study pave the road to unparalleled fundamental science and future applications of weak and strong light—matter interactions.

## 6. Methods: Sample Fabrication and Characterization [43]

The experimental samples consisted of silver films with a thickness of 40 nm deposited onto high-index prisms and relatively thick (2 µm to 3 µm) layers of a mixture of poly(methyl methacrylate) (PMMA) with Rhodamine 590 dye (Rh590) of different concentrations.

The high-index prisms were N-SF11 Right Angle Prisms purchased from Edmund Optics with a refractive index of 1.78 at 600 nm.

The data for the complex dielectric permittivity of silver was adopted from Johnson and Christy [44].

We assumed the index of refraction of PMMA to be *n* = 1.5 [45] within the range of 300 nm to 1000 nm.

Silver films were fabricated with the thermal vapor deposition technique using Nano 36 Thermal Evaporation Deposition System from Kurt J Lesker. The thickness of the films was measured with the stylus DekTak XT profilometer from Bruker.

The Rh590/PMMA films had the following concentrations of dye: 0 g/L (pure PMMA), 4 g/L, 16 g/L, 64 g/L, 128 g/L, 256 g/L, 512 g/L and 1260 g/L (pure Rh590), corresponding to masses of dye molecules in one liter (cubic decimeter) of solid compound, when solvent was evaporated. The latter parameters were evaluated based on values of densities of Rh590 [43] and PMMA [45].

In the process of the Rh590/PMMA film preparation, Rh590 was first dissolved in dichloromethane (DCM). The dissolution was performed in an ultrasonic bath at room temperature for ~5 min. Then, poly (methyl methacrylate) (PMMA, m.w. = 120,000 a.u.) was added, and the resulting mixture was placed for dissolution into the ultrasonic bath for ~15 min. As viscosity played an important role in the film formation, the amount of solvent was chosen accordingly to regulate the viscosity of the solution.

The solutions of Rh590/PMMA mixture were drop-casted, at 45°, onto silver films. The DekTak XT profilometer was used to estimate the thickness of the polymeric layers. In the thickness measurements, the films were scratched in several locations, and the results were averaged over the several test sites in each film.

The Lambda 900 spectrophotometer from PerkinElmer was used for the spectroscopic characterization in measuring transmission and reflection spectra. At a low concentration of Rhodamine 590 dye, the absorption spectrum shows the maximum at λ~530 nm and the shoulder at λ~490 nm. At higher dye concentrations, the shoulder becomes more pronounced. In the modeling, the dielectric permittivity of Rh590/polymer is modeled as a sum of contributions from two Lorentz oscillators [40] embedded in the PMMA host matrix [46]. Choosing the relative strengths, the spectral positions and the line-widths of Lorentzians as fitting parameters, this model well matched the experimental absorption spectrum.

The reflectance spectra of the Kretschmann geometry samples illuminated with p polarized light were measured as a function of the incidence angle in the spectrophotometer setup equipped with the 150 mm integrating sphere (Lambda 900 from PerkinElmer, Shelton, CT, USA), Figure 2.

Emission spectra were taken in the setup depicted in Figure 5 and Figure 6. For excitation, we used Q-switched frequency doubled Nd:YAG laser with the pulse duration t_pulse_ = 12 ns, at the wavelength of λ = 532 nm. The sample was placed on the rotation stage and oriented at the angle at which the SPP at λ = 532 nm was excited. The spontaneous emission of the dye molecules was collected via an optical fiber bundle connected to the entrance slit of the monochromator (MS 257 from Oriel/Newport) (Figure 5). In some studies, the emission spectra were measured in the spectrofluorometer setup (Fluorolog 3 from Jobin Yvon Horiba).

## Data Availability

Data underlying the results presented in this paper are not publicly available at this time, but may be obtained from the authors upon reasonable request.
